# Development of Polymer-Assisted Nanoparticles and Nanogels for Cancer Therapy: An Update

**DOI:** 10.3390/gels7020060

**Published:** 2021-05-17

**Authors:** Bibi Noorheen Haleema Mooneerah Neerooa, Li-Ting Ooi, Kamyar Shameli, Nuraina Anisa Dahlan, Jahid M. M. Islam, Janarthanan Pushpamalar, Sin-Yeang Teow

**Affiliations:** 1 Department of Medical Sciences, School of Medical and Life Sciences, Sunway University, Jalan Universiti, Bandar Sunway, Subang Jaya 47500, Malaysia; 16101248@imail.sunway.edu.my; 2 School of Health Sciences, International Medical University, 126, Jalan Jalil Perkasa 19, Bukit Jalil, Kuala Lumpur 57100, Malaysia; ooi.liting@student.imu.edu.my; 3 Department of Chemical and Environmental Engineering, Malaysia-Japan International Institute of Technology, Universiti Teknologi Malaysia, Kuala Lumpur 54100, Malaysia; kamyarshameli@gmail.com; 4 School of Science, Monash University Malaysia, Jalan Lagoon Selatan, Bandar Sunway, Subang Jaya 47500, Malaysia; nurainaanisa93@gmail.com (N.A.D.); jahid.islam@monash.edu (J.M.M.I.); pushpa.janarthanan@monash.edu (J.P.); 5 Monash-Industry Palm Oil Education and Research Platform (MIPO), Monash University Malaysia, Jalan Lagoon Selatan, Bandar Sunway, Subang Jaya 47500, Malaysia

**Keywords:** polymer, nanoparticles, nanogels, cancer therapy, mechanisms

## Abstract

With cancer remaining as one of the main causes of deaths worldwide, many studies are undergoing the effort to look for a novel and potent anticancer drug. Nanoparticles (NPs) are one of the rising fields in research for anticancer drug development. One of the key advantages of using NPs for cancer therapy is its high flexibility for modification, hence additional properties can be added to the NPs in order to improve its anticancer action. Polymer has attracted considerable attention to be used as a material to enhance the bioactivity of the NPs. Nanogels, which are NPs cross-linked with hydrophilic polymer network have also exhibited benefits in anticancer application. The characteristics of these nanomaterials include non-toxic, environment-friendly, and variable physiochemical properties. Some other unique properties of polymers are also attributed by diverse methods of polymer synthesis. This then contributes to the unique properties of the nanodrugs. This review article provides an in-depth update on the development of polymer-assisted NPs and nanogels for cancer therapy. Topics such as the synthesis, usage, and properties of the nanomaterials are discussed along with their mechanisms and functions in anticancer application. The advantages and limitations are also discussed in this article.

## 1. Introduction

Cancer remains one of the main causes of fatality in the world. In 2018 alone, 9.6 million cancer-related deaths were estimated [1]. The cancer type with the highest incidence rate were lung and breast cancer followed by prostate and colon cancer. The most fatal cancer type was lung cancer followed by stomach and liver cancer [1]. Anticancer drugs such as cisplatin, doxorubicin, paclitaxel, and 5-fluorouracil are commonly used for chemotherapy or used in combination with radiotherapy or tumor resection [2]. However, the development of resistant tumors and high toxicity of drugs towards the normal cells highly limit the use of these drugs [2]. Hence, the development of a novel therapeutic agent against cancer is needed.

Nanoparticles (NPs) are nano-size particles that have been greatly explored for various biomedical application such as antibacterial [3,4,5] and cancer therapy [6,7]. These NPs can be synthesized mainly through chemical and biological methods. Some of their advantageous properties which are suitable for various biomedical applications include their microscopic size for improved cell permeation, rich surface chemistry which allows diverse surface modification, as well as high stability and versatility, serving as therapeutic agents or nanocarrier for drug delivery. In recent years, NPs have been extensively studied for cancer therapy [6,8,9]. However, some of the limitations of NPs such as high toxicity and low solubilization have hindered their use for further development [10].

To overcome the abovementioned limitations, natural and synthetic polymers have been used to generate polymer-assisted NPs to be utilized as nanocomposites for several biomedical applications including cancer therapy [11,12] and tissue regeneration [13]. Various types of polymer-based NPs include core–shell NPs, polymersome, polyplex, and micelles can be fabricated via several methods such as emulsification, nanoprecipitation, electrospraying, and microfluidic technology. With the advent of the boosting development of nanotechnology in recent years, polymers have been exploited to improve the potency of NPs in cancer treatment following various engineering strategies. For instance, nanocomposites such as nanogels which are NPs composed of a crosslinked hydrophilic polymer network (also known as hydrogel) have been used as nanocarriers for anticancer drug delivery to the specific target site [14]. In addition, some of the nanogels could also reverse the toxicity of the NPs towards the target cell while retaining the anticancer potency [15]. In this review, various types of polymer-assisted NPs and nanogels, their promising features for cancer treatment, and the underlying mechanisms are discussed. We also attempt to discuss the potential limitations and challenges of developing these nanocomposites for clinical application.

## 2. Types of Polymer-Based Nanoparticles and Nanogels

### 2.1. Formulation of Polymeric Nanoparticles and Nanogels

**Figure 1:**
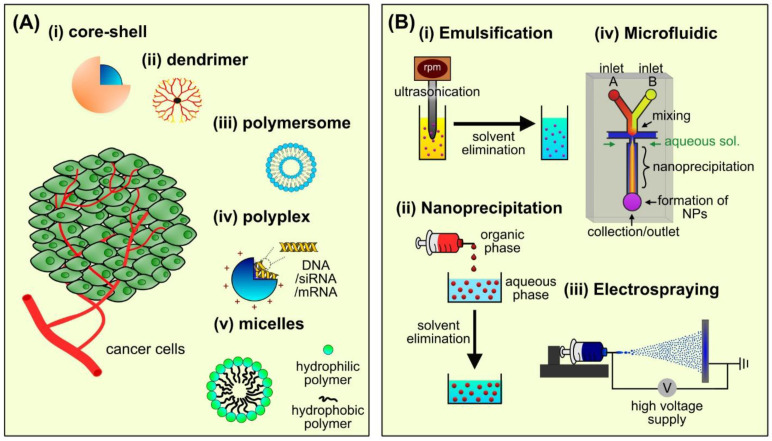
(**A**) Different types of polymer-assisted nanoparticles for cancer therapy (i) core–shell, (ii) dendrimer, (iii) polymersome, (iv) polyplex, and (v) micelle and (**B**) fabrication strategies used to synthesize the nanoparticles (i) emulsification, (ii) nanoprecipitation, (iii) electrospraying, and (iv) microfluidic technology.

Polymeric NPs are solid colloidal systems between 1 and 1000 nm. These NPs are known to be biocompatible, biodegradable, and cost-effective at an industrial level compared to most inorganic NPs. Figure 1A illustrates the different types of polymer-assisted NPs including core–shell NPs, polymersomes, polyplexes, and polymeric micelles fabricated using wide selections of natural and synthetic polymers. Depending on the fabrication strategies (Figure 1B), these polymeric NPs can easily form complexation with drugs, nucleic acids, or inorganic materials for specific cancer therapy [16].

#### 2.1.1. Core–Shell Nanoparticles

Core–shell polymeric NPs are biphasic nanomaterials made up of core (blue) and shell (brownish-orange) structures, as shown in Figure 1A(i). These NPs can be prepared from various biocompatible polymers such as silk fibroin, dextran, hyaluronic acid, polyethylene glycol, zwitterionic polymer, and polypeptides [17,18,19,20,21,22]. Previous studies reported that the core–shell structures could combine multiple functionalities within the layers, thereby forming the ‘smart’ nanocomposite fabrication strategy. Interestingly, Wu et al. [20] designed self-assembled polymeric-assisted NPs consisting of chitosan micelles loaded with SNX2112 (a synthetic heat shock protein 90 inhibitor) as the inner core. The crosslinking between cysteine and hyaluronan via disulphide linkages formed the outer shell. The active binding of hyaluronan to the CD44 receptor allowed the targeted delivery of the NPs to the breast cancer cells, followed by the release of drugs induced by pH and environmental stimuli. Zhang et al. [23] reported the controlled release of a conjugated bioactive biphenolic compound (honokiol) from core–shell NPs of zein-hyaluronic acid while the in vivo study showed enhanced antitumor and antimetastatic effects in the tumor-bearing mice.

#### 2.1.2. Dendrimer/Hyperbranched Polymer

Dendrimers are precisely synthesized macromolecules having a unique three-dimensional (3D) globular architecture consisting of a central core, internal dendritic structures, and peripheral functionalized surfaces. Dendrimers offer intriguing advantages such as surface functionalities, highly controllable shape depending on the number of repetitive units (branches), excellent biocompatibility, and ability to entrap higher payloads within the nanosized structure [24]. Interestingly, dendrimers have been considered ideal vectors for delivering anticancer drugs, genes, proteins, oligonucleotides, and peptides for cancer therapy [24,25]. These bioactive agents can be encapsulated within the cavities of internal dendritic structures or coupled through direct covalent conjugations (e.g., amide, ester, ortho-nitrobenzyl linkages) or cleavable linkers (e.g., pH, redox, photo, and enzyme-sensitive linkers) [25,26,27]. Toxicity of dendrimers contributed by the hydrophobic central core and highly cationic surface may hinder their biological applications. Studies reported that a strong interaction between highly cationic dendrimers (e.g., amine-terminated poly(amido-amine) (PAMAM) and polypropylenimine (PPI)) and negatively charged cellular membranes could lead to destabilization of cellular membranes and increased reactive oxygen species (ROS), eventually cell apoptosis. It was also reported that the toxicity level increased with the increase of dendritic branches [28]. In this case, smart dendrimers with tailored surface functionalities achieved through PEGylation, zwitterion, glycosylation, and targeting agent functionalization could produce safer and biocompatible nanoparticles with higher therapeutic efficacy [26,27,28,29,30].

#### 2.1.3. Polymersome

Polymersomes are stable bilayer vesicular systems prepared from amphiphilic copolymers that resemble the structure of natural liposome [31]. Studies reported different polymerization techniques to synthesize amphiphilic copolymers such as ring-opening polymerization (ROP), atom transfer radical polymerization (ATRP), and reversible addition-fragmentation chain transfer polymerization (RAFT) [31,32,33]. The amphiphilic nature of the block copolymers allows spontaneous self-assembly into high molecular weight aggregates. Compared to other polymeric NPs, polymersomes offer several advantages: readily adjustable membrane permeability, efficient delivery of hydrophobic, and hydrophilic drugs due to the hydrophobic outer layer, tuneable physicochemical properties by manipulating the thickness of the membrane, and high stability [31,32,33,34]. Kocere et al. [33] demonstrated the pH-responsiveness of their polyethylene glycol-*block*-poly (2-diisopropyl amino)ethyl methacrylate (PEG-PPDPA) polymersomes by releasing doxorubicin (DOX) only at low pH. They found that the cytotoxicity of DOX encapsulated in the polymersomes was reduced by 40-fold compared to the free DOX. Fluorescent imaging showed that most fluorescent-labelled PEG-PDPA polymersomes were accumulated in the tumor mass of zebrafish compared to the surrounding tissues. In another study, ultra-small superparamagnetic magnetic iron oxides (SPION) were generated via in situ chemical precipitation in the hydrophilic coronas of folic acid-polyglutamic acid-*block*-polycaprolactone polymersomes. As expected, the polymersomes-containing T_2_ contrast agent (ultra-small SPION) recorded ultrahigh T_2_ relaxivity at lowest iron (Fe) dosage. The fabricated magnetic polymersomes were then used in targeted cancer therapy and theranostic magnetic resonance imaging (MRI) application [32].

#### 2.1.4. Polyplex

Gene therapy is one of the promising treatments of cancers. The main objective of gene therapy is to introduce specific nucleic acids into the targeted cancerous cells or their surrounding cells to inhibit further cell growth and eventually cell death [35]. Ideally, appropriate delivery vehicles are required to transport these nucleic acids as they could not be uptake by the negatively charged plasma membranes due to their anionic characteristics. It has been found that cationic polymers could condense the nucleic acids to form polyplex NPs and deliver the material to target cells protected from the enzymatic degradation [16,35,36]. Gao et al. [37] prepared polyplex NPs through complexation of poly(amide-amine)-poly-(β-amino ester) hyperbranched copolymer (hPPC) with CRISPR/Cas9 recombinant plasmid. The polyplex NPs were designed to specifically target the HPV E7 oncogene of HPV-positive cervical cancer. Transfection experiments reported higher transfection efficiency of the fabricated polyplex NPs compared to the commercial transfection reagent and polyethylenimine (PEI) 25 kDa, the gold standard for non-viral vectors. In another study by Baghaei et al. [36], positively charged trimethyl chitosan (TMC) and several negatively charged polyelectrolytes (hyaluronate, alginate, dextran sulfate) were developed as the non-viral vectors of human SET1 (hSET1) antisense for cancer gene therapy. Their work demonstrated that the mixture of positively charged TMC and negatively charged hyaluronate produced the most optimized and non-cytotoxic polyplex NPs around 131 nm. In addition, they detected the accumulation of NPs at the target models both in in vitro and in vivo studies.

#### 2.1.5. Polymeric Micelles

Micelles are polymeric NPs formed via supramolecular self-assembly of amphiphilic block copolymers with distinct hydrophobic and hydrophilic properties. The structure of polymeric micelles is composed of a hydrophobic core and hydrophilic outer surface (shell) as illustrated in Figure 1A(iv) [38,39]. The hydrophobic core acts as a reservoir of poorly soluble anticancer drugs that protect them against the harsh biological microenvironment and rapid metabolism [40]. Micelles have a narrow size distribution (10–100 nm) and hydrophilic outer surface compared to other drug delivery nanocarriers, which enable long-time circulation in the bloodstream. Expanding the circulation time allows sufficient accumulation of drug-loaded micelles at the targeted sites for slow-acting cancer therapy [39,41]. Wu et al. [42] synthesized phenylboronic acid (PBA)-decorated methoxy polyethylene glycol-block-(poly-N-2-hydroxyethyl-aspartamide) (mPEG-b-PHEA/PBA (PPBA)) via self-assembly. The donor-acceptor coordination PBA moieties on the polymer backbones and two hydrophobic drugs (DOX and irinotecan (IR)) enhanced the concurrent loading of both DOX and IR. Significant tumor suppression was observed in vivo after treating with DOX and IR co-loaded polymeric micelles, demonstrating excellent synergistic anticancer efficacy. Barve et al. [43] loaded cabazitaxel into PEG-cholesterol micelles functionalized with enzyme-responsive peptide. They reported that the cabazitaxel-loaded micelles responded well to the overexpression of matrixmetaloproteinases (MMPs) by the prostate cancer cells, followed by cleaving the micellar structures which led to the release of cabazitaxel into the tumor microenvironment.

#### 2.1.6. Nanogels

Nanogels are three-dimensional (3D) hydrogels composed of crosslinkable hydrophilic polymer networks. Structural versatility of nanogels such as inherent porosity and high water retention allows exceptional drug loading capacities (more than 30% weight) owing to their enhanced colloidal and dispersion stabilities compared to other types of polymeric NPs such as micelles and polymersomes [44]. Interestingly, nanogels are unique due to having both hydrogels and NPs characteristics simultaneously, which made them great candidates for selective and targeted anticancer drug delivery. Apart from their distinct swelling behaviour, nanogels possessed high surface area, which indirectly enhances cargo bioavailability (i.e., drugs or proteins). They can also be further functionalized owing to the presence of various functional groups to respond to different physiological environments such as pH and temperature [45]. In this way, targeted drug delivery can be achieved, minimizing problems related to non-targeted drug accumulation and toxicity concern. For example, pH-responsive nanogels may consist of pendant acidic (-COOH), amino (-NH_2_), or other functional groups that ionize at a pH greater than pK_a_ of the polymeric network. In this instance, electrostatic repulsion between the chain of hydrogel’s structure causing an increased water uptake and swelling. The nanogels can be further functionalized to express altered hydrophobic and hydrophilic properties by introducing other functional groups such as cholesteryl (hydrophobic) and pullulan (hydrophilic) [46]. Meanwhile, Chang and Tsai [47] reported that the addition of sodium copper chlorophyllin (SCC) into their temperature-responsive polymer of poly(*N*-isopropylacylamide) (pNIPAAM) equipped the final nanogels with photothermal responsive properties. Although drug-inorganic NPs conjugates could easily permeate tumor vasculature due to enhance permeability and retention (EPR) effect, their colloidal instability and agglomeration problem could significantly hamper their continual use as drug nanocarriers [48]. In this case, immobilizing these inorganic NPs could help to combat the problems mentioned earlier. In fact, studies on immobilizing various inorganic NPs such as gold NPs, silver NPs, and graphene oxide along with drugs showed improved therapeutic efficiency [48,49,50].

### 2.2. Fabrication Strategies of Polymeric Nanoparticles

#### 2.2.1. Emulsification

Emulsification involves mixing the dissolved polymer phase into partially or completely immiscible liquid phase in the presence of surfactants. Surfactants reduce the surface tension between two liquid phases leading to stable emulsion droplets [51]. Typically, external forces such as stirring and sonication are required to break down the emulsion droplets into nanoemulsions. Emulsions are classified as oil-in-water (o/w) emulsion or water-in-oil (w/o) emulsion. Oil-in-water emulsion consists of the oil phase being dispersed in the form of droplets into a continuous water phase, and vice versa for water-in-oil emulsion. Generally, w/o emulsion has lower stability than o/w emulsion due to water droplets’ high mobility [51,52]. The formation of polymeric NPs via emulsification can be achieved either by emulsification-solvent evaporation or emulsification-solvent diffusion methods [51,53]. For instance, Szczęch and Szczepanowicz [54] prepared several NPs containing polycaprolactone (PCL), polylactic acid (PLA), and polylactide-co-glycolide (PLGA) using emulsification-solvent evaporation method. In this method, each polymer was dissolved in chloroform, followed by the addition of anionic oil-soluble surfactant and absolute ethanol (organic solution). Nanoemulsions were formed through the addition of organic solution into an aqueous solution containing polycation. Subsequently, the solvent residues were evaporated either by increasing the temperature under reduced pressure or continuous magnetic stirring to obtain dispersed solid NPs. They found that the surface of solid polymeric NPs (100 to 200 nm) can be easily functionalized for cancer applications including bioimaging, as well as passive and magnetic targeting. In emulsification-solvent diffusion, partially water-miscible solvents such as ethanol, acetone, and ethyl acetate were used to dissolve the polymers and bioactive materials (organic phase) followed by addition into the surfactant-containing aqueous phase under continuous magnetic stirring [55,56]. The addition of excess water triggered solvent diffusion from the dispersed droplets to the external aqueous phase, causing the aggregated oil droplets to form colloidal NPs. In addition, evaporation or filtration methods can remove the solvent residues and form solid NPs [51,53]. Feng et al. [56] evaluated several parameters influencing the fabrication of fisetin encapsulated PLA nanoparticles via emulsification-solvent diffusion. They observed that the NP’s physicochemical properties were influenced by the surfactant’s concentration (poloxamer 188), o/w phase ratio (1:7 showed the best ratio), acetone-to-ethyl acetate ratio, and fisetin-to-PLA mass ratio.

#### 2.2.2. Nanoprecipitation

Nanoprecipitation is a one-step and direct method widely used to produce nanospheres and nanocapsules. This method is also known as the solvent displacement method. It involves the mixing of two miscible solvent systems which result in spontaneous precipitation. The exposure of the organic phase (dissolved biomaterials) to the aqueous phase induces precipitation of materials in nanoscale particles. This method produces NPs with well-defined morphology and narrow size distribution (~200 nm), which is highly desirable in cancer treatment [51,57]. Despite the rapid NPs formation, nanoprecipitation method resulted in poor encapsulation efficiency as low as 20% [57]. Furthermore, nanoprecipitation is mostly used to encapsulate hydrophobic drugs [53]. Encapsulating hydrophilic drugs using this method yielded lower drug loading capacity compared to hydrophobic drugs. This is due to the higher premature leakage of hydrophilic drugs in the aqueous solution during the nanoprecipitation process [58]. Besides, selecting appropriate solvents is highly crucial as solvent residues could induce a cytotoxic effect which reduces the NPs efficiency. Almoustafa et al. [59] stated that solvent residues from dimethylformamide (DMF), dimethyl sulfoxide (DMSO), and chloroform would be challenging to be removed using solvent evaporation technique. Instead, these residues can be removed via dialysis. However, the synthesized NPs are exposed to agglomeration problems with prolonged incubation in the aqueous solution as dialysis is a time-consuming method.

#### 2.2.3. Electrospraying

Electrospraying uses electrical force to fabricate polymeric NPs. The general set-up of electrospraying is illustrated in Figure 1B(iii) consisting of a syringe loaded with a precursor solution that passes through a metallic needle, a syringe pump to control the flow rate, a high voltage power supply, and a grounded collector. Under a high electric field, the precursor solution formed a curvature of drop surface known as Taylor cone at the end of the needle. As the electrical field increases and exceeds charged liquid’s surface tension, smaller charged droplets are formed via Coulomb repulsion forces and travels to the grounded collector. During this process, the solvent evaporates from the droplets forming solid NPs as they are captured by the grounded collector [60,61,62]. Formation of uniform NPs using electrospraying method is controlled by several parameters such as polymer solutions (conductivity, concentration, viscosity), solvent properties (dielectric constant, miscibility, viscosity, vapor pressure), and electrospraying conditions (voltage, flow rate, distance between the tip of the needle and the grounded collector) [63,64]. Ghaffarzadegan et al. [65] modified the conventional electrospraying technique by connecting two feed systems. The first syringe consists of berberine (hydrophobic drug) dissolved in ethanol/DMF mixture, whereas the second syringe was loaded with PLA dissolved in chloroform/DMF mixture. Electrical force was applied to the needle tip connecting both feed systems allowing the formation of core–shell NPs with berberine as the core surrounded by PLA matrix (shell). They reported that the cytotoxicity and cellular uptake of the core–shell NPs were significantly higher than the free berberine. A more advanced electrospraying method such as a multiplex nozzle system offers the fabrication of complex NPs at an industrial scale compared to conventional electrospraying. The formation of uniform NPs relies on various external factors, including geometries of the needle tip (circular, triangular, or hexagonal), nozzle-substrate configuration, and flow rate. For instance, Parhizkar and co-workers reported a higher formation of uniform NPs produced by four electrospraying nozzles arranged in circular configuration [66].

#### 2.2.4. Microfluidic Technology

Microfluidic represents a miniaturized technology of manipulating liquids at nano- or microscales. This advanced technology has been widely used in various biomedical applications such as NPs fabrication, cell or NPs separation, vesicle isolation, and point-of-care testing [53,65,66,67,68,69,70]. Therefore, microfluidic technology is another promising approach to fabricate polymeric NPs for cancer therapy. Compared to other fabrication strategies, microfluidic offers enhanced reproducibility and scalability at an industrial scale while controlling batch-*to*-batch uniformity [70]. Fabrication of bulk NPs using a microfluidic system commonly adopted either (1) droplet-based flow focusing method that produces microparticles or (2) rapid mixing and nanoprecipitation method to produce smaller NPs. In rapid mixing and nanoprecipitation-microfluidic system, the polymer precursor stream injected from both inlets is forced to form a narrow stream along the central channel due to the high flow rates of a parallel aqueous stream. This causes nanoprecipitation of the polymeric materials leading to the formation of monodisperse NPs with narrow size distribution (10–100 nm) [57,70]. Chiesa et al. [71] investigated the operational parameters of modified microfluidic-assisted nanoprecipitation using a mathematical modelling. They reported that the total flow rate (TFR) and flow rate ratio (FRR) operating parameters influenced the formation of PLGA NPs. It was observed that as the TFR increased and FRR decreased, the system yielded well-defined PLGA NPs (<200 nm). Meanwhile, Wang and colleagues utilized the microfluidic technology to design conjugated polymeric NPs functionalized with tumor-homing peptide as photothermal therapy agents. The synthesized nanoparticles (52 nm) exhibited excellent photothermal performance to trigger an immune response in vitro and in vivo leading to suppression of tumor growth in mice [72].

#### 2.2.5. Preparation of Nanogels

**Figure 2:**
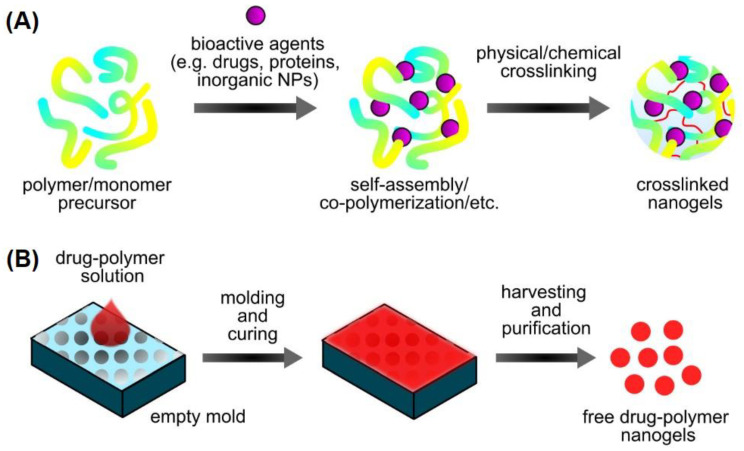
Overview of nanogels synthesis routes through (**A**) conventional physical/chemical crosslinking from natural and synthetic polymers. (**B**) particles replication in non-wetting template (PRINT) from customized matrices with controllable shape, size and composition.

Nanogels are synthesized from both natural and synthetic polymers or peptides sequences. The functional groups of these polymers (e.g., carboxyl, amine, hydroxyl, thiol, and sulfo groups) are responsible for crosslinking the polymeric chains via physical interactions or chemical crosslinking to form 3D nanogels (Figure 2A). Meanwhile, the residual functional groups along the crosslinked networks contribute to the nanogels’ swelling characteristic [45]. In physical crosslinking, the polymeric networks bond through various non-covalent interactions such as hydrogen bonding, ionic interaction, hydrophilic-hydrophilic, and hydrophobic-hydrophobic interactions. These interactions force the polymeric chains to collapse, leading to the formation of 3D polymeric nanostructures. On the other hand, chemical crosslinking involves the co-polymerization of polymer or monomer chains and covalent crosslinking in the presence of chemical crosslinkers. Aldehydes, epichlorohydrin and glutaraldehyde are commonly used chemical crosslinkers having reactive groups to form linkages between the polymeric chains [45,73]. Although chemical crosslinking offers stable nanogels’ formation, the use of highly toxic solvents and crosslinkers such as glutaraldehyde during the reaction synthesis may hinder its continual use in biomedical applications. Radiation-engineered nanogels allows the facile synthesis of nanogels in the absence of potentially toxic chemical compounds (e.g., solvents, crosslinkers, surfactants, and initiators), thereby eliminating additional purification steps [74]. The only substrates involve in the reaction are polymers and water. Formation of instantaneous hydroxyl radicals (·OH) and hydrogen (H) atoms along the polymer chains from the radiolysis of water upon direct irradiation (e.g., pulsed electron beam or gamma irradiation) on an aqueous polymer solution. The radiation-engineered nanogels networks originate from the inter- and intramolecular crosslinking of the generated polymer radicals. In this case, shorter polymer chains are prone to undergo intermolecular crosslinking, whereas intramolecular crosslinking favors longer polymer chains [75,76]. Irradiation technique may offer simultaneous nanogels synthesis and sterilization, provided the same irradiation dose applied to achieve both desired properties and sterility, thus attractive for various biomedical uses [77]. Studies reported formation of radiation-engineered nanogels using a wide variety of polymers such as chitosan, polyacrylic acid (PAA), polyvinyl alcohol (PVA), polyethylene oxide (PEO), poly(*N*-vinylpyrrolidone) (PVP), among others [74,75,77]. Recently, particles replication in non-wetting template (PRINT) emerged as an advanced nanogels fabrication. The PRINT technique as shown in Figure 2B facilitates the fabrication of monodisperse nanogels using various customized matrices with controllable shape, composition, and size. This technique is highly advantageous as it allows the loading of delicate active agents and complex biomolecules [78].

### 2.3. Types of Polymers for Polymeric Nanoparticles Formation

#### 2.3.1. Natural Polymers

Natural polymers are commonly used to prepare bioactive polymeric NPs. For example, hyaluronic acid, chitosan, alginate, and dextran have been used as biomaterials to construct NPs for cancer treatments [79,80]. Natural polymers exhibit biocompatible, biodegradable, non-cytotoxic, and non-immunogenic properties which are highly desirable in cancer therapy [81,82]. Hyaluronic acid is a highly hydrophilic polymer and a major component of the extracellular matrix. Targeting and inhibiting overexpressed cancer-specific receptors on the surface of cancer cells such as CD44 and receptor for HA-mediated motility (RHAMM) is one of cancer therapy strategies. Studies reported that hyaluronic acid could target the cancer cells by binding to these receptors following the internalization of hyaluronic acid-based NPs into the cancer cells and exert the anticancer effect [80,83]. Similar strategy has also been adopted for targeted delivery of cancer-targeting small-interfering RNA (siRNA) and anticancer drugs or compounds such as doxorubicin and curcumin [80,82,83,84].

Chitosan is a linear polysaccharide composed of β-(1-4)-d-glucosamine (deacetylated unit) and *N*-acetyl-d-glucosamine (acetylated unit). Chitosan is synthesized from chitin’s deacetylation, an abundant natural polymer extracted from crustaceans’ exoskeleton such as shrimps, crabs, and crayfish [81]. Similarly, chitosan has been used to construct polymeric NPs for cancer treatment. For instance, Sorasitthiyanukarn et al. [85] studied the uses of chitosan/alginate NPs encapsulating curcumin diglutaric acid for oral delivery. They reported that these loaded NPs showed enhanced anticancer activity in vitro. Besides, chitosan is a cationic polymer due to the presence of amine groups in its polymeric chain. The amine groups allow further functionalization or modification of chitosan to fabricate complex NPs with bioactive materials for bioimaging and targeted drug and gene deliveries [84].

Alginate is a hydrophilic polymer with an anionic charge extracted from marine bacteria and brown seaweed. Due to its contrasting charge, alginate can easily form functional NPs with cationic polymers such as chitosan through polyelectrolyte complexation [79,86]. The formation of alginate-chitosan NPs via ionic interaction between carboxyl groups of alginate and amine groups of chitosan was reported by Sohail and Abbas [79]. The alginate-chitosan NPs were loaded with a drug, amygdalin, and the sustained release of amygdalin over 10 h of incubation was shown. In another study, Tawfik et al. [86] prepared DOX-loaded alginate functionalized-upconversion NPs. The smart NPs showed a promising potency of anticancer drug delivery and its use for near-infrared (NIR) imaging. Dextran is a complex branched glucan with more than 50% of (α-1,6)-linkages in its major chains. The side chains of dextran consist of several percent of (α-1,3) while (α-1,4) and (α-1,2) linkages make up the least number in its structure [87,88]. Studies reported that dextran could be used as the main biomaterial synthesizing of pH and redox dual responsive NPs. For instance, DOX and lipoic acid were successfully conjugated to polyaldehyde dextran which acted as nanocarriers for camptothecin. The release of camptothecin was regulated by the pH and redox potential stimuli [87].

#### 2.3.2. Synthetic Polymers

Contrary to natural polymers, synthetic polymers are synthesized from reactive monomers via polymerization reactions. It is relatively easier to control the synthetic polymers’ reproducibility, scalability and mechanical performance compared to naturally derived polymers. Besides, synthetic polymers can be facilely modified and functionalized depending on the desired biomedical applications [81,89]. Despite being synthetic, these polymers or their derivatives can be degraded into non-toxic oligomers or monomers, which could then be eliminated from the biological system via normal metabolic pathways [89]. In this respect, PEI, polystyrene (PS), PLA and PLGA are commonly used synthetic polymers for the fabrication of polymeric NPs. PEI is a cationic polymer favored as a non-vector for the delivery of nucleic acids. However, the non-degradable property of native PEI hinders its further application in cancer therapy. In clinical settings, the accumulation of polyplex NPs containing PEI in the organs and bloodstream could potentially cause high cytotoxicity leading to the failure of the therapeutic NPs in vivo [90,91,92]. This limitation can be overcome by developing PEI derivatives through modification or functionalization to enhance their degradability. For example, the cytotoxicity of multifunctional NPs consisting of oleylamine—modified disulfide-containing PEI was significantly lower than the branched PEI (25 kDa) [91].

Another cationic synthetic polymer, PS is commonly used to generate biocompatible polymeric NPs. Studies showed that PS derivatives such as polystyrenesulfonic acid and maleimide-modified polystyrene could enhance the cellular uptake and exert low cytotoxicity towards healthy cells [93,94]. On the other hand, PLA is a FDA-approved synthetic polymer which possesses bioabsorbable, biodegradable and biocompatible properties [95]. PLA has been used to develop smart polymeric NPs in various forms using different strategies, including bioactive-loaded NPs, bioactive-conjugated NPs, and copolymerization with other polymers to express multi-responsive behaviour [40,95,96]. For instance, Dariva and colleagues developed amphiphilic light-sensitive NPs consisting of PEG-PLA copolymers conjugated with 1,2-bis(2-hydroxyethylthio) ethylene. This work demonstrated the feasible loading of the model drug, DOX into the complex polymeric NPs for localized drug delivery [40]. Contrary to PLA, hydrophilic PEG is among the most developed polymeric NPs that has been widely used for various biomedical applications. PEG-based NPs are intrinsically less toxic with the ability to reduce cytotoxicity of cationic polymers to shorten renal clearance, thereby extends circulation time in blood [97]. Chemically, PEG has two hydroxyl (-OH) end groups which can conveniently crosslink with other polymers or anticancer drugs through various covalent linkages. PEG can serve as a coating material on anticancer drugs encapsulating nanoparticles through PEGylation technique. Notably, the terminal -OH end groups can be facilely replaced with various reactive functional groups (e.g., amine, aldehyde, carboxymethyl, succinimido succinate, mesylate, and bromo), thus enhance the stability and solubility of PEG-conjugated anticancer drugs NPs in vivo as well as optimizing their drug efficacy [98,99]. Lastly, PLGA is a copolymer of PLA and polyglycolic acid (PGA). PLGA is another FDA-approved synthetic polymer as it could degrade biologically into non-toxic lactic acid and glycolic acid through hydrolysis of the ester backbone. For this reason, PLGA has gained attention for its use in cancer therapy, particularly in drug delivery, imaging, and diagnostics [89,96]. Interestingly, various types of targeting moieties can be introduced to PLGA allowing the fabrication of hybrid and targeted NPs which are useful as drug delivery systems. For example, small oligonucleic acids (aptamers) specific for heparinase (HPA) were conjugated to PEG-functionalized PLGA NPs followed by paclitaxel loading. This enabled high cellular uptake by HPA-expressed triple-negative breast cancer cells, followed by the release of paclitaxel for tumor cell killing [100].

#### 2.3.3. Peptide-Polymer Conjugates

Presently, various peptide-polymer conjugates have emerged as promising cancer therapeutic agents and imaging by virtue of their biocompatibility, biodegradability, and tunable properties. Researchers have explored the possible conjugation between peptide precursors and polymers to form smart NPs for sequential targeting or enhance the efficacy of encapsulated payloads in vivo [101,102]. Most recently, CC-9 peptide conjugated to poly(acrylamide-co-methacrylic acid) nanogels showed enhanced co-localization with SW-48 colon cancer cell lines to augment colon cancer targeting abilities [103]. To date, various peptides (e.g., papain, albumin, arginine-glycine-aspartic acid (RGD), and azido-bi-functionalized peptides) have been used in the development of peptide-polymer conjugates [101,104,105,106]. Studies reported that these peptide-polymer NPs provides new opportunities to generate hybrid NPs with attractive properties such as tumor targeting, stimuli- and microenvironmental responsiveness while minimizing off-target toxicity [101,104].

## 3. Functionally Active Nanoparticles for Cancer Therapy

### 3.1. Specific Feature of Polymers for Cancer Therapy

One of the key reasons of utilizing polymers for NPs-based cancer therapy is to improve the cancer cell killing while providing the safe and stable delivery of the anticancer agents. Polymers are suitable candidates as they possess various bio-compatible properties including high flexibility for material modification, high biodegradation rate and chemical inactiveness. Surface modifications can be performed on the polymer to render them more biocompatible through physical or chemical methods such as chemical and plasma treatment, ion implantation, and UV irradiation [107,108]. The high degradation rate of certain polymers such as PGA and PCL ensured the safety of the use of material and protect the environment [109]. Organic polymer with high biocompatibility can be used for tissue engineering applications involving conventional cell growth, production of hybrid tissues, and artificial organs. Self-healing is also a desirable trait where the materials have the ability to repair the cellular damage [110].

Other favored properties that polymers may possess are high hydrophobicity and adhesion potential. The high hydrophobicity is required to prevent the coagulation of blood during the blood circulation. However, depending on the need, the polymer can be engineered and turned into water-soluble form. The cell adhesive properties of the polymers towards a specific or multiple cell type or tissue can also be manipulated [110]. This characteristic is usually required in polymers that are used for drug delivery. Other properties such as diverse topology and chemistry also favor the use of polymers for cancer therapy. They can be either originated from natural, semi-natural or synthetic sources and appear in various shapes such as lined, branched, graft, crosslinked, dendron, block, star-shape, or microspheres [111]. Polymers that are used for drug delivery can be stimuli responsive where drug release can be controlled either by temperature, UV, or pH. Additionally, stimuli responsive polymers can also be used to control cell adhesion in order to stimulate gene expression or enzyme function [112,113].

### 3.2. Engineering Strategies of Nanogels for Cancer Therapy

**Figure 3:**
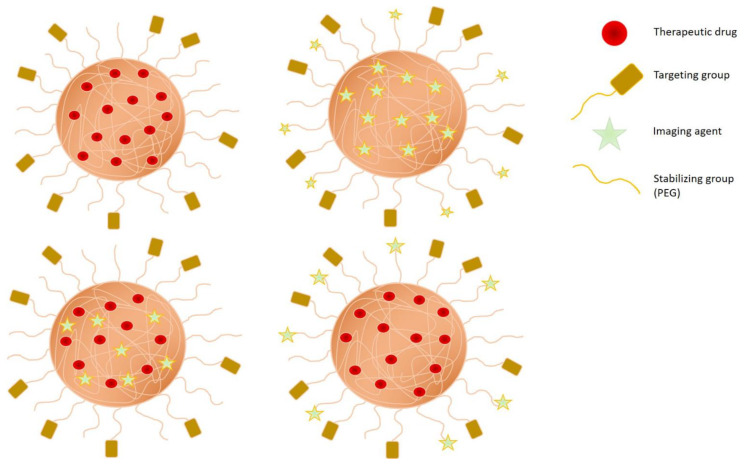
Illustrations of the design of nanogels for treatment, diagnosis, and theranostic in anticancer applications.

Polymeric NPs in different designs can be used in various anticancer applications including treatment, diagnostic and theranostic purposes (Figure 3). Biocompatibility, stability, biodegradability, ease of modification, and cost-effectiveness are key consideration in using polymeric NPs for cancer therapy. The cancer tissues can be targeted either passively or actively. Passive targeting can be achieved by the enhanced permeability and retention effect (EPR) in which the polymeric nanocarriers tend to passively accumulate at the tumor site. To improve this effect, PEGylation of the polymeric NPs particularly in the range of 100 to 200 nm in size can increase the circulation time as well as prevent the opsonization. On the other hand, active targeting can be accomplished by the incorporation of aptamer, protein, peptides, antibodies, nucleic acid, or small molecule onto the surface that can specifically bind to the desired receptor or surface ligand. Hence, the polymeric NPs can actively transport the drug or other therapeutic agent to the tumor site. Similarly, PEGylation can also be incorporated to render the active targeting more effective [114,115].

**Figure 4:**
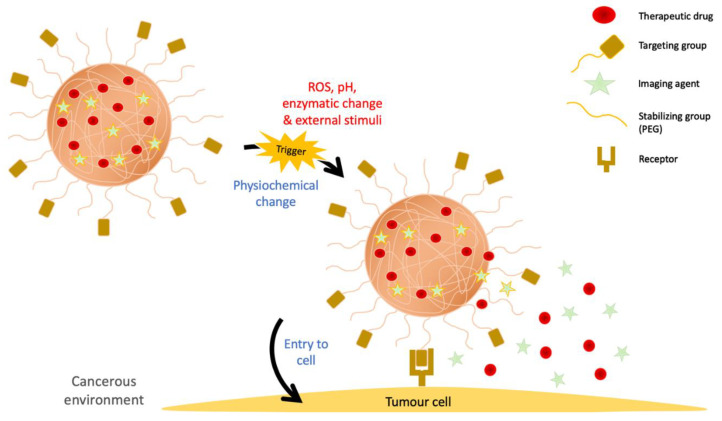
Schematic representation of how nanogels are used in theranostic approach. Physiological changes are triggered by cancer cell surrounding environment (ROS level, pH, enzymatic changes, and external stimuli) to release drugs and imaging agents.

Naturally, tumor cells have uncontrolled and hyperactive proliferative activity. This forms a unique surrounding known as ‘tumor microenvironment’ for the tumor cells to gain support and continuously grow and expand. The common conditions of the microenvironment often include lowered or acidic pH, increased level of reactive oxygen species (ROS), and overexpressed tumor-supporting enzymes (Figure 4). These conditions can sometimes affect the potency of the polymeric NPs and the delivered drugs, but recently, these conditions have been employed to activate and generate ‘smart’ polymers in turn which could benefit the cancer therapy. For example, pH-responsive polymers can be activated at the low pH of pH 4.5 to pH 6 (typically pH at tumor site) and release the therapeutic drugs in a controlled manner. Similarly, ROS-responsive polymer with disulphide-based structure is stimulated in the presence of high level of ROS while the enzyme-responsive polymer responds to the accumulation of oncogenic enzymes such as cathepsins and MMPs for maximized drug delivery and cancer cell killing. Furthermore, external stimuli such as light radiation, temperature, and magnetic field can also be used for the controlled release and ultimately enhance the cancer therapy [114].

### 3.3. Polymeric Nanomaterials Targeting Various Cancers

Polymers such as PEG is one of the most used material in generating NPs. PEG is known to be highly versatile and with the addition of other polymers, it could efficiently enhance drug delivery. For example, DOX-IR780-loaded PEG-PCL NPs improved the delivery of DOX and IR780 to bladder cancer [116] while NOS-mPEG-PLGA NPs [117], ANZ-PLNPs [118], DOX-loaded PEGylated pH-sensitive NPs [119] improved the delivery of drugs to breast cancer. 5FU-Chrysin-PLGA-PEG-PLGA NPs were found to improve the delivery of the drug 5-FU and Chrysin in colon cancer [120]. Two PEG NPs, DOX-VER-MPEG-PLA NPs [121] and MET-PLGA-PEG NPs [122], were able to improve the drug delivery to ovarian cancer with DOX-VER-MPEG-PLA NP being able to efficiently encapsulate the drugs for delivery. Other than being able to deliver drugs to gastrointestinal, hepatic, and blood cancer, Nisin-loaded PLA-PEG-PLA NPs can protect nisin and was able to sustain the release of nisin comparing to free nisin [123]. Linoleic acid-conjugated SN38 (LA-SN38)-loaded PEO-PBO NPs increased drug loading and entrapment efficiency for LA-SN38 to colon cancer and similarly controlled the release of LA-SN38 [124]. Epidermal growth factor receptor (EGFR)-targeted CCPD and DOX-loaded lipid polymeric NPs had a faster release of DOX and delivered the drugs to NSCLC cells more potently [125].

PLGA, a co-polymer made from poly lactic acid and poly glycolic acid was also used to produce NPs [126]. Afatinib or miR-loaded PLGA NPs were able to prevent the degradation of afatibin and miR, thereby resulting in the stable and potent delivery of these compounds in colon cancer [127]. UT-PLGA and UTPCL exhibited efficient delivery of drugs but UT-PLGA had a slightly better drug loading and could target prostate cancer cells effectively [127]. In addition to the PLGA NPs, Cur-PLGA NPs was able to stabilize curcumin in the presence of light and improve curcumin serum stability when compared to free curcumin [128].

Chitosan NPs are known to have good stability, low toxicity, and can be administrated through many routes of administrations [129]. MRT/CBZ-TPC-CS NPs and QCT-CS NPs drastically improved the drug delivery in both breast and lung cancers [130]. The enhanced drug delivery of quercetin to the target site was attributed to the improved encapsulation and the sustained release of the drug [131]. Similarly, 3A.1-loaded pH-sensitive chitosan NPs [132] and Cs-CPT NPs [133] specifically targeted and delivered the drugs to colon cancer cells. SVCSNPs was shown to exhibit controlled release of SV, hence increasing the accumulation of drug in intracellular compartments of hepatic cancer cells [134].

Albendazole-loaded polyurethane NPs [135], NVA NPs [136], DOX-loaded estradiol-conjugated hypoxia-responsive NPs [137], and Bortezomib-loaded HPLA-BT NPs [138] could efficiently target and suppress breast cancer tumors. More specifically, DOX-loaded estradiol-conjugated hypoxia-responsive NPs was able to target the hypoxic areas of positive estrogen-receptors breast cancer microtumors [137]. Cur-loaded phenylboronic acid-containing framboidal NPs was able to improve the chemical stability of Cur and maintained the release of Cur under physiological conditions [139]. In lung cancer treatment, platinum–curcumin complexes were loaded into pH and redox dual-responsive NPs which showed effective intracellular drug release [140] while sorafenib (SF)-loaded cationically-modified polymeric NPs released the drugs through aerosolization of NPs [141]. Naringenin-loaded hyaluronic acid decorated PCL NPs also facilitated drug delivery in NSCLC cells [142]. Gemcitabine (GEM) NPs conjugated with linoleic acid (GEM NPs) have a high drug load, able to control the release of drug, and improve the intracellular uptake in thyroid cancer [143]. DTX-loaded NPs assisted the drug delivery to ovarian cancer [144]. Drug efficacy of benznidazole was improved in BNZ-SA-Chol-PMMA NPs in colon, cervical, and hepatic cancers [145].

In addition to improving drug delivery and sustained drug release, most polymer-assisted NPs possess specific actions against tumor cells including cancer cell apoptosis induction, antiangiogenecity, and antiproliferation (Table 1). NPs such as DOX-IR780-PEG-PCL-SS NPs [116], ABZ-Polyurethane NPs [135], QCT-CS NPs [131], DOX-loaded pH sensitive PEGylated NPs [119], Cur-loaded phenylboronic acid-containing framboidal NPs [139], NAR-HA@CH-PCL NPs [142], DTX-loaded Ecoflex^®^ NPs [144], DOX-VER-MPEG-PLA NPs [121], and Cur-PLGA NPs [128] were able to inhibit the tumor growth and reduce the tumor weight. In addition, ABZ-Polyurethane NPs was able to induce apoptosis in the cancer cells while Cur-PLGA NPs exhibited antiangiogenic activity in colon cancer [128]. MET-PLGA-PEG NPs also induced nuclei fragmentation and apoptosis in the treated cancer cells [122]. Other NPs which were reported to induce apoptosis in cancer cells are Nos-mPEG-PLGA NPs [117], NVA polymeric NPs [136], ANZ-PLNP NPs [118], 3A.1-loaded pH-sensitive NPs [132], CS-CPT-NPs [133], 5FU-Chrysin-PLGA-PEG-PLGA NPs [120], and Gemcitabine-loaded NPs conjugated with linoleic acid [143]. Nos-mPEG-PLGA NPs [117] has antiangiogenic properties while NVA polymeric NPs [136] inhibited cellular proliferation.

Furthermore, Afatinib or miR-loaded PLGA NPs was able to target and penetrate more effectively into the cells which increased the sensitivity of cells to afatinib [146]. PteCUR@PSPPN enhanced the anti-metastatic activity by blocking the PI3K/AKT signal transduction pathway [140]. Sorafenib (SF)-loaded cationically-modified polymeric NPs enhanced the inhibition of cell migration, reduced cell survival, and inhibited the clonogenic formation [141]. Table 1 summarizes various types of polymer-assisted NPs used for cancer therapies and their functions.

Currently, there are many nanomaterials undergoing clinical trials in hopes of finding the next alternatives in cancer therapy. Table 2 shows various anticancer nanomaterials that are currently undergoing clinical trials who are still recruiting test subjects. Magnetic NPs coated with antibodies such as EpCAM and CD52 as markers are used to filter out tumor cells. EpCAM antibodies targets prostate, colon, lung, or pancreatic cancer. While CD52 is used as a marker towards lymphoma or leukemia [147]. A cetuximab-loaded NP generated from ethycellulose is a pH-responsive nanocomposite in which cetuximab is stimulated to release at pH above 6.8 while the drug remains bound within the NPs at pH lower than 6.8 [148]. For treatment monitoring, an immuno-tethered lipoplex nanoparticle biochip is being tested on patients with B-cell lymphoma and it is also being tested for the detection of cancer relapse in patients [149]. A type of nanoparticle targeting brain metastasis is made from gadolinium and polyxiloxane as it has great theronostic properties [150]. Quantum dots NPs that are decorated with veldoreatide is used to deliver anticancer drugs for suppression of breast cancer cells and to also assist in the bioimaging of the cancer cells [151]. A PLGA nanoparticle containing a tumor antigen (NY-ESO-1) and IMM60 to induce anti-tumor responses in New York Esophageal Squamous Cell Carcinoma-1 positive patients [152].

A clinical trial using USPION, or known as ultrasmall superparamagnetic iron oxide nanoparticles is made from ferrotan. It is use to detect any lymph node metastasis of solid tumors before operation with the help of magnetic resonance imaging (MRI) in pancreatic cancer [153]. Two clinical trials using superparamagnetic iron oxide nanoparticles (SPION) is currently ongoing for two types of cancer, breast and liver (hepatocellular) cancer. In the clinical study for breast cancer, SPION is use to trace any delayed sentinel lymph node dissection, while in the study for hepatocellular cancer, SPION is used to increase the safety of the liver by detecting and avoiding high levels of radiation after radiotherapy [154,155]. Three studies using NBTXR3, a nanoparticle that is made from hafnium oxide for the improvement of radiation sensitivity of tumor cells. One study focuses on the destruction of pancreatic cancer cells through radiation therapy [156], while the other two studies target head and neck squamous cell cancer by improving the effectiveness of radiation therapy [157,158].

Albumin-bound nanoparticles with rapamycin (nab-rapamycin) are undergoing clinical trials which targets advance nonadipocytic soft tissue, solid tumors, glioma, and glioblastoma. Nab-rapamycin are used as a combinational therapy with other available drugs to improve anticancer effects. In the clinical trial aiming at nonadipocytic soft tissue, it is used along with pazopanib hydrochloride which may halt the tumor growth by blocking growth enzymes [159]. To target solid tumors, nab-rapamycin is used as a combinational therapy with temozolomide, and irinotecan to evaluate the drug response towards the solid tumors [160].

Another chemotherapy drug that is widely used that are albumin bounded are albumin-bounded paclitaxel (nab-paclitaxel). Nab-paclitaxel is being tested to improve the patient survival by combining with gemcitabine instead of gemcitabine only as a treatment for pancreatic cancer [161]. Other trials on pancreatic cancer using nab-paclitaxel are used as a combinational therapy with other drugs to improve the anticancer effect as nab-paclitacel is used to halt tumor growth by killing, arresting cell division, or by preventing metastasis [162,163,164,165]. These functions and the usage of nab-paclitaxel can also be seen in many other ongoing clinical trials targeting different type of cancers such as triple negative breast cancer, biliary tract cancer, liver bile duct cancer, B-cell non-Hodgkin lymphoma, NSCLS, and esophageal cancer [166,167,168,169,170,171,172].

**Table 2:** Anticancer nanomaterial currently in clinical trial.

Nanoparticle	Polymer	Function of Polymer	Cancer Type	Year	Status	Reference
Magnetite nanoparticles	Magnetic nanoparticles, coated with antibodies	The coating of the magnetic nanoparticles consist of antibodies such as epithelial cell adhesion molecule (EpCAM) and CD52 as markers to attempt to remove tumor cells from the blood.	prostate, colon, lung or pancreatic cancer and lymphoma or leukemia	2017–current	Recruiting	[147]
Cetuximab nanoparticles	Ethylcellulose, decorated with somatostatin analogue	Release drug at only above pH 6.8. Holds Cetuximab at pH 1.5.	Colon Cancer	2018–current	Recruiting	[148]
Immuno-tethered lipoplex nanoparticle (ILN) biochip	Immuno-tethered lipoplex	To monitor treatment response and to detect relapse in patient.	B-cell lymphoma	2018–current	Recruiting	[149]
Gadolinium-chelated polysiloxane based nanoparticles	Gadolinium-chelated polysiloxane	Has great theronostic properties by radiosensitization and diagnosis by multimodal imaging.	Brain metastases	2019–current	Recruiting	[150]
Quantum dots nanoparticles	Cds/ZnS core–shell type quantum dots with carboxylic acid, decorated with veldoreotide	Using veldoreotide as a somatostatin analog to deliver anticancer drugs to target and for bioimaging of the cancer cells.	Breast Cancer	2019–current	Recruiting	[151]
PLGA nanoparticle	PLGA	PLGA nanoparticles are minimal in toxicity and is used for drug delivery for anti-tumor immune response.	New York Esophageal Squamous Cell Carcinoma-1 (NY-ESO-1) positive cancers	2021–current	Recruiting	[152]
Ultrasmall Superparamagentic Iron Oxide nanoparticles (USPION)	Ferrotran	To detect lymph node metastases of solid tumors with the assistance of MRI.	Pancreatic Cancer	2017–2021	Recruiting	[153]
Superparamagentic Iron Oxide nanoparticles (SPION)	Iron Oxide	To trace delayed sentinel lymph node dissection.	Breast Cancer	2020–current	Recruiting	[154]
Superparamagentic Iron Oxide nanoparticles (SPION)	Iron Oxide	Increase the safety of liver after stereotactic body radiotherapy by assisting in the detection and the avoidance of high levels of radiation.	Hepatocellular carcinomas	2020–current	Recruiting	[155]
Hafnium Oxide-containing nanoparticles NBTXR3	Hafnium Oxide	To target cancer cells for destruction through radiation therapy.	Pancreatic Cancer	2020–current	Recruiting	[156]
Hafnium Oxide-containing nanoparticles NBTXR3	Hafnium Oxide	To improve sensitivity of tumor cells to radiation therapy.	Recurrent/Metastatic Head and Neck Squamous Cell Cancer	2021–current	Recruiting	[158]
Hafnium Oxide-containing nanoparticles NBTXR3	Hafnium Oxide	Using NBTXR3 to improve the effectiveness of radiation therapy.	Head and Neck Squamous Cell Cancer	2021–current	Recruiting	[157]
Albumin-bound Rapamycin nanoparticle (Nab-rapamycin)	Albumin	Along with pazopanib hydrochloride, it may block cell growth enxymes which in turn halts the growth of tumor cells.	Advance Nonadipocytic Soft Tissue Sarcomas	2019–current	Recruiting	[159]
Albumin-bound rapamycin, temozolomide, and irinotecan nanoparticles	Albumin	To evaluate the drug response as a combinational therapy.	Solid Tumors	2017–current	Recruiting	[160]
Albumin-bound Rapamycin nanoparticles (Nab-Rapamycin)	Albumin	To evaluate the effect of Nab-rapamycin alone or in combination with various drugs in patients.	Glioma and Glioblastoma	2018–current	Recruiting	[173]
Paclitaxel Albumin-bound nanoparticles (Nab-Paclitaxel)	Paclitaxel Albumin-bound combining with gemcitabine, and cisplastin with high dose of Ascorbic Acid	To improve survival of paclitaxel albumin-bound nanoparticle with gemcitabine comparing to only gemcitabine.	Pancreatic cancer	2017–current	Recruiting	[161]
Albumin-bound Paclitaxel nanoparticle (Nab-paclitaxel)	Albumin	As combinational drug therapy with cisplastin and gemcitabine.	Pacreatic Adenocarcinoma	2019–current	Recruiting	[162]
Albumin-bound Paclitaxel nanoparticle (Nab-paclitaxel)	Albumin	Nab-paclitaxel is able to stop tumor growth by killing, arrest cell division, or by preventing it from metastasis.	Metastatic Pancreatic Cancer	2020–current	Recruiting	[163]
Albumin-bound Paclitaxel nanoparticle (Nab-paclitaxel)	Albumin	Nab-paclitaxel is able to stop tumor growth by killing, arrest cell division, or by preventing it from metastasis.	Metastatic Pancreatic Cancer	2020–current	Recruiting	[164]
Albumin-bound Paclitaxel nanoparticle (Nab-paclitaxel)	Albumin	As combinational drug therapy.	Pancreatic Cancer	2020–current	Recruiting	[165]
Albumin-bound Paclitaxel nanoparticle (Nab-paclitaxel)	Albumin	As combinational drug therapy.	Triple Negative Breat Cancer	2018–current	Recruiting	[166]
Albumin-bound Paclitaxel nanoparticle (Nab-paclitaxel)	Albumin	Nab-paclitaxel is able to stop tumor growth by killing, arrest cell division, or by preventing it from metastasis.	Triple Negative Breast Cancer	2020–current	Recruiting	[174]
Albumin-bound Paclitaxel nanoparticle (Nab-paclitaxel)	Albumin	Nab-paclitaxel is able to stop tumor growth by killing, arrest cell division, or by preventing it from metastasis.	Biliary Tract Cancer	2018–current	Recruiting	[167]
Albumin-bound Paclitaxel nanoparticle (Nab-paclitaxel)	Albumin	Combinining nab-paclitaxel, gemcitabine, and cisplastin to halt growth of tumor cells.	Liver Bile Duct Cancer	2018–current	Recruiting	[168]
Nab-paclitaxel/Rituximab-coated nanoparticle	Albumin	Combining Nab-paclitaxel to halt growth by killing or stopping the groth of tumor cells, while using rituximab may affec the growth and spreading of tumor cells.	Reccurent or refraxtory B-cell non-Hodgkin lymphoma	2019–current	Recruiting	[169]
Albumin-bound Paclitaxel nanoparticle (Nab-paclitaxel)	Albumin	To evaluate the drug response as a combinational therapy.	Non-squamous non-small cell lung cancer (NSCLS)	2020–current	Recruiting	[170]
Albumin-bound Paclitaxel nanoparticle (Nab-paclitaxel)	Albumin	As combinational drug therapy with cisplastin and capecitabine	Esophageal cancer	2020–current	Recruiting	[171]
Albumin-bound Paclitaxel nanoparticle (Nab-paclitaxel)	Albumin	As combinational drug therapy with cisplastin and sinitilimab.	Esophageal cancer	2020–current	Recruiting	[172]

## 4. Mechanism of Anticancer Action

Mechanism of the anticancer effect of biopolymer-based NPs is similar to other forms of NPs. However, because of several active groups’ presence, biopolymer-based NPs offer more diversity and binding opportunities. In cancer therapeutics, NPs usually act as nanocages and are equipped with anticancer drugs inside the cage. In this regard, the potency of the nano-therapeutics mainly depends on successful unloading of the drug cargo into the target site, which is mediated by three consecutive pathways, i.e., targeting, cellular uptake, and drug release.

### 4.1. Targeting

**Figure 5:**
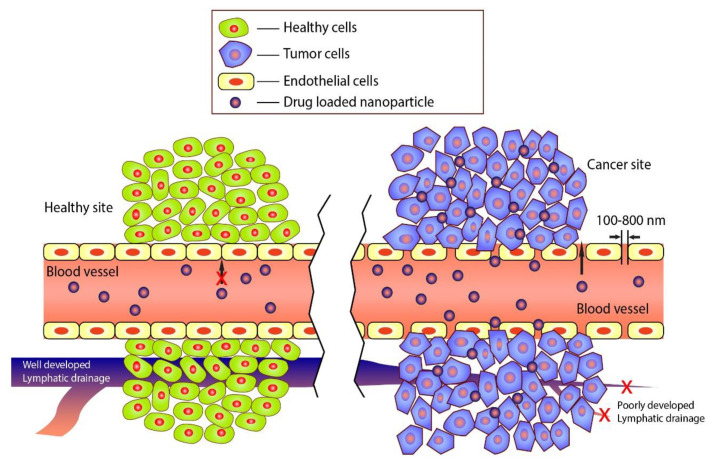
Passive targeting mediated by Enhanced Permeability and Retention (EPR) effect. The (**left side**) represents the healthy tissue which has blood vessels made of tightly bound endothelial cells. Whereas the endothelial cells of the blood vessel in cancer tissue comprises loosely bound endothelial cells (**right side**), leaving pores to facilitate NPs permeability.

In cancer therapeutics, the most challenging part is to guide the anticancer agents into the targeted cancer site, as non-specific targeting often leads to severe damage to the subject’s healthy tissue or organ. Nanotechnology has brought a new dimension in the cancer-targeting system, mediated by passive and active targeting. Passive targeting works based on the size-dependent permeability of nanoparticles. In the rapidly growing cancer tissue, angiogenesis occurs at an exceptionally high rate, compromising the blood vessels’ rigidity and thus enables passive targeting. In this mechanism, NPs can penetrate through the larger pores of the leaky blood vessels of tumor tissues. However, they cannot pass through the comparatively tight blood vessels of a healthy organ [175]. Besides, tumor tissues have an underdeveloped lymphatic system. Therefore, an uptake of NPs cannot leak out from the targeted site, thereby increasing drug concentration in the cells [176] (Figure 5).

However, organs like liver and spleen also possess larger pores in the blood vessels, making them vulnerable to passive targeting. In this regard, nowadays, nanoparticles are often equipped with active targeting ligands. Any moiety with selective affinity to bind with any unique/overexpressed receptor in the cancer cells can act as targeting ligand and is usually introduced by chemical conjugation with the NPs. Thus, combining passive and active targeting, these NPs accumulate at much higher concentration in cancer tissues than other healthy tissues and organs; therefore, increasing the therapeutic dose in the cancer cell and reducing side effects [175,176].

Worldwide extensive cancer research has revealed a large variety of active targeting sites. A range of molecules from protein to small peptides, glycosaminoglycan like hyaluronic acid, vitamins like folic acid, antibodies or antibodies fragments, aptamers or even carbohydrates or polysaccharides are found very effective for targeting [177,178,179]. Transferrin, a serum glycoprotein, has been widely used as the transferrin receptor is overexpressed in many types of metastatic cancer cell [180,181]. Besides, bombesin peptide, transferrin, arginine–glycine–aspartic acid (RGD) peptide, and NR7 peptide bind with overexpressed gastrin-releasing peptide receptor, integrin αvβ3 receptor and EGFR respectively on the tumor cell surface [182,183]. Glycosaminoglycan like hyaluronic acid has target specificity to CD44 receptor which is overexpressed in many types of cancer cell surface including colon, breast, head and neck, prostate, lung, and gastric cancers [184]. On the other hand, folate binding protein (FBP) is found in dividing cell’s surfaces as folate is an essential component for DNA synthesis and replication. Because of the rapid growing behavior, cancer cells overexpress FBP (folate receptor). Especially, breast, cervical, lung, ovarian, colorectal, kidney, and brain cancers overexpress it to a great extent, making folate an excellent targeting ligand for these cancer types [185].

### 4.2. Cellular Uptake

**Figure 6:**
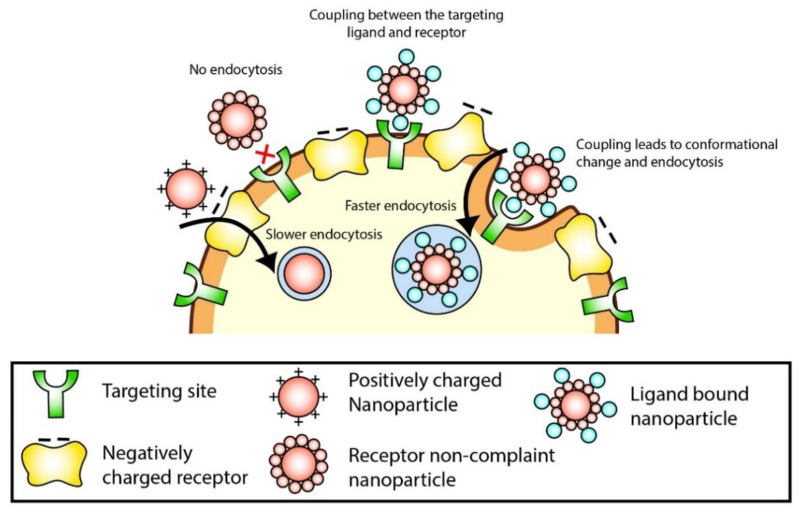
Ionic charge and receptor-mediated endocytosis of drug-loaded nanocomposite.

Cellular uptake efficiency is very critical for a successful nano-therapeutic. Cellular uptake of the NP depends on various factors like particle size, charge, hydrophilicity, presence of ligand. It is expected that the nanoparticles will be internalized by the cancer cells before the particles release their drug load. However, in case of external stimuli-responsive system, drugs may be forced to be released near the cancer tissue before the particles are internalized. This process increases drug concentration in the cancer tissue to a great extent compared to the other parts of the body and reduces the side effects. However, there is always a chance for the drugs to enter back in the systemic circulation in the released form, leading to adverse impacts. Therefore, the higher the cellular uptake in the cancer cells, the lesser chance for side effects. Cellular uptake for nanoparticles is often termed as endocytosis which is a complex energy-dependent and actin-mediated process. In this case, nanoparticles are internalized by either clathrin-coated pits or caveolae based on their particle size. These pathways are activated when a NP component (could be a NP building block or a targeting ligand) binds with corresponding cell surface receptor (Figure 6). In the clathrin-mediated pathway, particles ranging from 60 to 200 nm transported into the cells through the vesicles formed when clathrin-coated pits invaginate into the cell. On the other hand, particles larger than 200 nm are transported by specialized lipid rafts known as caveolae. In this caveolae pathway, caveolae interact with many signalling-associated proteins, such as G-protein–coupled receptors, tyrosine kinases receptors, and steroid hormone receptors, leading to bulkier formation invagination in the cell membrane [186].

Cellular uptake is also closely related to NPs charge and hydrophilicity. Positively charged NPs electrostatically interact with negatively charged (sulphate-containing) proteoglycans in the cell membrane and transported into the cell (Figure 6). Moreover, less hydrophilic or hydrophobic NPs can diffuse into the cells due to their lipid-binding property. However, non-specific binding in the systemic circulation can alter the surface property of a NP. Thus, the NPs may act differently in vivo compared to the in vitro experiments. Upon introducing the drug encapsulated NPs to the gastrointestinal tract in vivo, it creates a defence mechanism against the transition of NPs into the bloodstream. The pharmacokinetics of the NPs throughout the body is by absorption, distribution, metabolism, and excretion. The potential interaction is between the surface of the NPs and the bloodstream component, especially from the immune system. However, potential aggregation and surface absorption of blood plasma proteins form a “corona” that significantly contributes to the nanoparticles’ biological response. Thus, accumulation of the NPs expected at the targeted organs such as kidney, liver, and spleen. This could lead to oxidative stress, inflammation, and eventually to cell death at the targeted organ. Harmful nanoparticles need to be identified by assay methods such as endotoxin and lactate dehydrogenase signalling a cell death and oxidative stress for exposing biomarkers of induced-cellular damaging NPs. Newly developed NPs are required evaluation and judgement to understand the NPs threat to health before manufacturing and supply to consumers [187,188].

### 4.3. Drug Release

Drug release enables the therapeutic effect of the NP-based drug delivery system. In biopolymer-based NPs, the most critical factors for the drug release efficiency are hydrophilicity and degradation behavior. Besides, these NPs can be tailored to response against internal or external stimuli which can modulate their drug release. When the hydrophilicity of the NP is the rate-limiting factor, the hydrophilic segments start swelling in contact with the aqueous environment and eventually release entrapped drugs into the external environment. On the other hand, when degradation is the primary criterion for the drug release, the NPs undergo structural deformation and cleavage before releasing the trapped drugs. Hydrolysis, oxidation, or enzymatic degradation are the primary mechanisms for biopolymer-based NPs degradation.

In the case of stimuli-responsive drug release, physical, thermal, chemical, or biochemical stimuli can alter the conformation or structural composition of the NPs through decomposition, polymerization, isomerization, or supramolecular aggregation triggering the drug release. Light, temperature, electric fields, magnetic force, and ultrasound are examples of external stimuli. In contrast, changes in pH, the stress in target tissues, and ionic strength are the examples of internal stimuli [189]. Because of the oxygen shortage due to under-developed vascularization and fast-growing nature, tumor cells are forced to follow anaerobic pathways to meet their energy demand. This leads to high levels of acidic by-products like lactic acid and reductive agents like glutathione which form acidic and reductive microenvironment in the tumor tissue. NPs can be designed to undergo rapid degradation in low pH or reductive environments, enabling targeted drug release into the tumor tissue [190]. Another effective strategy is to incorporate an overexpressed enzyme (specifically in tumor cells) into the nanoparticle. In this case, the engineered nanoparticle is more likely to have increased degradation rate inside the tumor cells than the healthy cells and thus delivers more anticancer drugs into the tumor [191].

In case of external stimuli like temperature, UV, infrared or ultrasound responsive NPs, these stimuli reduce the stability of the nanoparticle and thus induces the release of the encapsulated drug. Fabricating nanoparticles conjugating with iron particles is another effective strategy to accumulate the NPs into the targeted tumor tissue by applying external magnetic forces [192,193].

## 5. Limitation and Challenges

Continuous studies on polymeric NPs and nanogels are being done to improve the cancer therapy. A list of advantages and limitations for cancer therapy are tabulated in Table 3. One of the key advantages is the modifiable physicochemical properties, which can increase the NPs circulation time in the bloodstream with enhanced bioavailability. This is to ensure the sustained release of bioactive materials in the polymeric NPs. Due to the ease of processability and modification, these NPs can be easily modified into various structures as discussed above. This property is crucial as it protects bioactive materials from degradation, which maintains their therapeutic effect. Despite the attractive benefits, potential toxicological problems due to the slow degradation rate expressed by certain synthetic NPs limit their further use in clinical application. There is also a growing concern about the safety of nanocomposites which interact with the tissues/organs. It is critical to understand the clearance time of the NPs in the biological system [194].

The development of safe and efficient polymer-assisted NPs and nanogels remains challenging for clinical translations. Therefore, further studies and optimizations are of paramount importance. Nano-bio interaction is one of the bottlenecks of clinical translations. The engineered polymeric NPs will instantaneously interact with surrounding biomolecules upon entering the complex biological system. This causes the formation of protein ‘corona’ on the surface of the NPs. Research found that this corona could alter the polymeric NPs properties and functions such as biodistribution, stability, pharmacokinetics, immune system, and toxicity [195,196]. Furthermore, numerous in vitro and in vivo animal studies have been done to understand the polymeric NPs mechanisms on specific cancer treatments. However, it is very challenging to translate these data into complex human biological systems and disease heterogeneity.

## 6. Conclusions

Increasing traction is gained towards the development of polymeric NPs and nanogels for various applications in cancer management including therapy and diagnostics. Following the discovery of new polymers with beneficial properties for cancer therapy, the new development of such nanocomposites is swift and the number of tumor-suppressing polymeric NPs and nanogels has drastically increased. Various types of cancer-targeting polymeric NPs and nanogels, their origin, synthesis method, anticancer actions and their underlying mechanism are discussed in this review. More importantly, recent update on the anticancer NPs and nanogels, and their target actions are also been discussed. To date, myriads of such NPs are under pre-clinical investigation and only one polymeric NPs has entered clinical trial. This is mainly due to the several issues that hinder the further development including lack of potency in vivo and toxicity issues. Further investigations are needed to revise the fabrication strategies to modify and improve the polymeric NPs and nanogels to resolve the abovementioned issues.

## Figures and Tables

**Table 1:** Various types of polymeric nanomaterials targeting different cancers in pre-clinical testing.

Nanoparticle	Polymer and Additives	Function of Polymer	Drug/Anticancer Compound	Cancer Type	Tested Model	Target Action	Year	Reference
Doxorubicin-IR780-PEG-PCL-SS NPs (DOX IR780-PEG-PCL-SS NPs)	PEG-PCL-SS	Drug delivery	Doxorubicin	Bladder Cancer	MB49 cells (Mouse, C57BL/Icrf-a’)	NIR laser-controlled drug release and imaging guidance for chemo-photothermal synergistic therapy reduce tumor size and inhibit growth	2020	[116]
Albendazole-loaded polyurethane NPs (ABZ-polyurethane NPs)	Polyurethane	Compatible to ABZ, better drug delivery	Albendazole	Breast Cancer	MCF-7, MDA-MB-231 cells	Apoptosis, increase ABZ anticancer potency	2020	[135]
Noscapine-loaded mPEG-PLGA NPs (NOS-mPEG-PLGA-NPs)	mPEG-PLGA	Anticancer effect of Noscapine improved when encapsulated in nanoparticles compared to free form	Noscapine	Breast Cancer	4T1 cells, 4T1 in BALB/c	Antiangiogenic, apoptotic effects	2020	[117]
mertansine (MRT) or cabazitaxel (CBZ) loaded TPC–CS NPs (MRT/CBZ-TPC-CS NPs)	Chitosan (CS) + tetraphenylchlorin (TPC)	Increase drug loading	Mertansine/Cabazitaxel	Breast Cancer	MDA-MB-231, MDA-MB-468 cells	MRT or CBZ had higher cytotoxic effect compared to free drug	2020	[130]
*A. absinthium* extract loaded polymeric nanoparticles (NVA-AA)	*NIPAAM-VP-AA*	Drug delivery	*Artemisia absinthium* extract	Breast Cancer	MCF-7, MDA MB-231 cells	Induces cytotoxicity, inhibition of cellular proliferation, induction of apoptosis	2020	[136]
Bortezomib (BTZ) loaded PNPs of HPLA-BT NPs	HPLA-BT	Drug delivery, higher drug load	Bortezomib	Breast Cancer	MCF-7 cells	Higher cytotoxic effects of DL (drug loaded) -HPLA-BT PNPs and significant anticancer activity	2020	[138]
Anastrozole loaded PEGylated polymer–lipid hybrid nanoparticles (ANZ -PLNPs)	PEG and lipid	stable encapsulated system with a high percentage of entrapment efficiency	Anastrozole	Breast Cancer	MCF-7 cells	Induction of apoptosis	2020	[118]
Estradiol-conjugated hypoxia-responsive polymeric nanoparticles encapsulating doxorubicin	PLA_17000_-PEG_2000_-Estradiol	targeted delivery into the hypoxic niches of estrogen-receptor-positive breast cancer microtumors	Doxorubicin	Breast Cancer	MCF7 cells	Higher cytotoxicity of targeted polymersomes in hypoxia compared to in normoxia	2020	[137]
quercetin loaded chitosan nanoparticles (QCT-CS NPs)	Chitosan	Better drug delivery, enhanced encapsulation efficiency and sustained release property	Quercetin	Breast Cancer	MDA-MB-468 cells	Cytotoxicity, decrease tumor growth	2018	[131]
				Lung Cancer	A549 cells			
DOX-loaded PEGylated therapeutic nanosystem for pH-sensitive release	PEG	releasing the drug in a controlled manner at acidic pH, increasing efficacy compared to doxorubicin in solution	Doxorubicin	Breast Cancer	MDA-MB-231 cells	Better anti-tumor activity, inhibits cell proliferation	2020	[119]
				Lung Cancer	A549, H520 cells			
3A.1-loaded pH-sensitive chitosan nanoparticles	naphthyl-grafted succinyl chitosan (NSC), octyl-grafted succinyl chitosan (OSC), and benzyl-grafted succinyl chitosan (BSC)	delivering anticancer drugs to the targeted colon cancer sites	Andrographolide analog	Colon Cancer	HT-29 cells	significantly lower IC50 than free drug and promotes apoptosis	2018	[132]
Linoleic acid conjugated SN38 (LA-SN38)-loaded NPs (EBNPs)	PEO-PBO diblock copolymer	EBNPs had high drug loading efficiency and entrapment efficiency for LA-SN38, release behaviour of EBNPs was slow and sustained	Linoleic acid conjugated SN38	Colon Cancer	HCT-116, HT-29 cells	Growth inhibitory effects, EBNPs promotes the uptake in cancer cells. EBNPs had prolonged blood circulation time.	2019	[124]
Cur-loaded phenylboronic acid-containing framboidal nanoparticles	PBAAM, PEGAM, MBAM	Improved chemical stability of Cur and sustained release under physiological conditions	Curcumin	Colon Cancer	HT-29 cells	Antiangiogenic, reduced tumor weight	2019	[139]
Chondroitin sulphate functionalized campththecin-loaded polymeric nanoparticles (CS-CPT-NPs)	Chitosan	Targeted drug delivery	Campththecin	Colon Cancer	CT-26 cells (Mouse, BALB/c)	significantly improved the anti-colon cancer activities, promote apoptosis effects	2019	[133]
Afatinib or miR- loaded polylactic-co-glycolic acid surrounded by PEG-lipids (shell modified with ligand R and pH-sensitive CPP H) nanoparticles (Afatinib or miR-loaded PLGA NPs)	PLGA	Protect Afatinib and miR, improve drug delivery	Afatinib/miR	Colon Cancer	Caco-2 cells	pH-responsive characteristics to increase the sensitivity of colon cancer cells to afatinib.	2019	[146]
5-FU-Chrysin-loaded PLGA-PEG-PLGA nanoparticles (5FU-Chrysin-PLGA-PEG-PLGA NPs)	PLGA-PEG-PLGA	Improve the functional delivery efficacy of 5-FU and Chrysin in cancer	5-FU, Chrysin	Colon Cancer	HT-29 cells	Apoptosis, growth inhibitory effects	2020	[120]
Simvastatin (SV) chitosan nanoparticles co-crosslinked with tripolyphosphate and chondroitin sulfate (SVSChSNPs)	Chitosan co-crosslinked with tripolyphosphate and chondroitin sulfate	Control the release pattern of SV. Particle size and positive surface charge of NPs enhances the accumulation of SV in intracellular compartments.	Simvastatin	Hepatic Cancer	HepG2 cells	enhanced the cytotoxicity of SV against HepG2 cells owing to its enhanced cellular uptake. ChS improved oral bioavailability	2020	[134]
Naringenin-loaded Hyaluronic acid (HA) decorated PCL NPs (NAR-HA@CH-PCL-NP)	PCL	Drug delivery	Naringenin	Lung cancer	A549 cells	Cytotoxic effect and active targeting of NAR-HA@CH-PCL-NP. Further treatment with NAR-HA@CH-PCL-NP was found effective in tumor growth inhibitory effect against urethane-induced lung cancer in rat	2018	[142]
EGFR-targeted LPNs loaded with CDDP and DOX	EGF-PEG-DSPE	Target drug delivery, faster release of DOX from LPNs than CDDP.	Doxorubicin	Lung Cancer	A549 cells	Improved anticancer activity with lower toxicity. Drug-loaded LPNs improved cytotoxicity	2019	[125]
platinum–curcumin complexes loaded into pH and redox dual-responsive nanoparticles (PteCUR@PSPPN)	mPEG-SS-PBAE-PLGA	control intracellular release, synergistic anticancer effects	Platinum–curcumin	Lung Cancer	A549 cells	Synergistic anticancer effects, enhanced anti-metastatic activity	2019	[140]
sorafenib (SF)-loaded cationically-modified polymeric nanoparticles (NPs)	PLGA	aerosolization efficiency for pulmonary delivery	Sorafenib	Lung Cancer	A549 cells	enhanced cell migration inhibition, reduction in cell survival, inhibition in the formation of colonies	2020	[141]
*Uncaria tomentosa* extract (UT)-PLGA & UTPCL	PCL and PLGA	Better drug delivery—UT-PLGA nanoparticles showed higher drug loading	*Uncaria tomentosa* extract	Prostate Cancer	LNCaP, DU145 cells	UT-PLGA showed higher cytotoxicity towards DU145 cells, UTPCL showed higher cytotoxicity against LNCaP cells	2019	[127]
Gemcitabine (GEM) NPs conjugated with linoleic acid (GEM NPs)	Linoleic acid	high drug-load, controlled release, improved intracellular uptake	Gemcitabine	Thyroid Cancer	B-CPAP, FTC-133 cells	Enhanced cytotoxic activity, induces apoptosis	2020	[143]
Ecoflex^®^ NPs loaded with DTX (DTX-NPs)	PEG 6000	Targeted drug delivery	Docetaxel	Ovarian Cancer	SKOV-3, MDA-468 cells	Increase antitumor efficacy, enhanced cellular uptake.	2018	[144]
DOX-verapamil/MPEG-PLA nanoparticles (DOX-VER-MPEG-PLA)	MPEG-PLA	co-delivery system –efficiently coencapsulate verapamil and chemotherapeutic agents.	Doxorubicin, Verapamil	Ovarian Cancer	A2780, SKOV3 cells	Tumor suppression	2018	[121]
Metformin-loaded PLGA-PEG nanoparticles (MET-PLGA-PEG NPs)	PLGA-PEG	Improve drug delivery	Metformin	Ovarian Cancer	SKOV3 cells	Increased nuclei fragmentation and amount of apoptotic cells induced by MET-NPs, enhance ani-cancer effects	2018	[122]
Curcumin (Cur)- loaded Polymeric poly(lactic-co-glycolic acid) (PLGA) nanoparticles (Cur-PLGA NPs)	PLGA	Stabilize curcumin in the presence of light, improved serum stability compared to free curcumin	Curcumin	Ovarian Cancer	SKOV3 cells	Cytotoxic effects on tumor cells upon irradiation at a low intensity inhibit tumor growth	2019	[128]
Nisin-loaded PLA-PEG-PLA nanoparticles	PLA-PEG-PLA	Better protection and sustained release for nisin	Nisin	Gastrointestinal Cancer	AGS, KYSE-30 cells	Higher cytotoxic effect in nisin-loaded NPs, increase cell growth reduction when comparing to free nisin	2018	[123]
				Hepatic Cancer	Hep-G2 cells			
				Blood Cancer	K562 cells			
Benznidazoles (BNZ)-loaded cationic polymeric nanoparticles (NPs) (BNZ-SA-Chol-PMMA NPs)	cationic polymethyl-methacrylate (PMMA) NPs	Improves drug efficacy	Benznidazoles	Colon Cancer	HT-29 cells	BNZ-NPs improved anticancer effect	2019	[145]
				Cervical Cancer	HeLa cells			
				Hepatic Cancer	Hep-G2 cells			

**Table 3:** Advantages and limitations of nanoparticles and nanogels.

Advantages	Disadvantages/Limitations
Adjustable physicochemical properties Protection of bioactive materials (nucleic acids, proteins, drugs, etc.) against in vivo degradation Controlled and targeted drug delivery Improving patient’s adherence towards prescribed medication Longer circulation time with improved bioavailability High drug loading capacity High stability	Potential toxicology problems due to the slow degradation rate of certain synthetic polymers Batch-*to*-batch variation for NPs synthesized from natural polymers Safety on polymeric NPs-tissues/organs interaction Accumulation of polymeric NPs in tissues/organ Difficult to scale-up complex NPs (e.g., multi stimuli polymeric NPs, multiple loading of drugs, etc.)

## Data Availability

Not applicable.
